# HEALTH AND WELL-BEING OF FAMILY CAREGIVERS FOLLOWING HOSPICE USE

**DOI:** 10.1093/geroni/igac059.1417

**Published:** 2022-12-20

**Authors:** Rebecca Utz, Michael Hollingshaus, Katherine Ornstein, Djin Tay, Caroline Stephens, Ken Smith

**Affiliations:** University of Utah, Salt Lake City, Utah, United States; University of Utah, Salt Lake City, Utah, United States; Johns Hopkins, University, Baltimore, Maryland, United States; University of Utah, Salt Lake City, Utah, United States; University of Utah, Salt Lake City, Utah, United States; University of Utah, Salt Lake City, Utah, United States

## Abstract

Some patterns of hospice use are potentially disruptive and stressful for both patient and family. Using a decedent sample of 17,320 hospice users from the Utah Population Database, linked to the health records of spouse and first-degree relatives (child, parent, sibling, Mean=3.36 per decedent). Stroke decedents had higher odds of delayed admission (i.e., <7 days of hospice), while those with dementia and COPD had higher odds of extended use (i.e., >6 months of hospice), compared to cancer decedents. Family members, especially spouses, who experienced delayed admission or extended use had higher odds of hospitalization and increased use of antidepressants, both prior to and following the death, compared to those with more normative hospice use (6 months or less, typical of cancer patients). The hospice experience has both immediate and lasting consequences on the family; eligibility criteria for the hospice benefit may need to be modified by admitting diagnosis.

